# Risk Factors for Delayed Entrance into Care after Diagnosis among Patients with Late-Stage HIV Disease in Southern Vietnam

**DOI:** 10.1371/journal.pone.0108939

**Published:** 2014-10-16

**Authors:** Suresh Rangarajan, Hoang Nguyen Bao Tram, Catherine S. Todd, Tran Thinh, Van Hung, Pham Thanh Hieu, Tran My Hanh, Khong Minh Chau, Nguyen Danh Lam, Pham Tri Hung, Gary West, Donn Colby

**Affiliations:** 1 FHI360 Vietnam, Ho Chi Minh City, Vietnam; 2 Asia-Pacific Regional Office, FHI 360, Bangkok, Thailand; 3 Ho Chi Minh City Provincial AIDS Committee, Ho Chi Minh City, Vietnam; 4 District 8 Preventive Medicine Center, Ho Chi Minh City, Vietnam; 5 An Giang Provincial AIDS Center, Long Xuyen, An Giang, Vietnam; 6 Tinh Bien Hospital, An Giang, Vietnam; 7 Can Tho Provincial AIDS Center, Can Tho, Vietnam; 8 Thot Not Outpatient Clinic, Can Tho, Vietnam; 9 FHI360 Vietnam, Hanoi, Vietnam; 10 Center for Applied Research on Men and Health, Ho Chi Minh City, Vietnam; 11 SEARCH, Thai Red Cross AIDS Research Center, Bangkok, Thailand; David Geffen School of Medicine at UCLA, United States of America

## Abstract

**Background:**

We surveyed HIV patients with late-stage disease in southern Vietnam to determine if barriers to access and service quality resulted in late HIV testing and delays from initial diagnosis to entry into HIV care.

**Methodology:**

196 adult patients at public HIV clinics with CD4 counts less than 250 cells/mm3 completed a standardized questionnaire. We used multivariate analysis to determine risk factors for delayed entry into care, defined as >3 months time from diagnosis to registration.

**Results:**

Common reasons for delayed testing were feeling healthy (71%), fear of stigma and discrimination in the community (43%), time conflicts with work or school (31%), did not want to know if infected (30%), and fear of lack of confidentiality (27%). Forty-five percent of participants delayed entry into care with a median CD4 count of 65 cells/mm3. The most common reasons for delayed entry were feeling healthy (51%), fear of stigma and discrimination in the community (41%), time conflicts with work or school (33%), and fear of lack of confidentiality (26%). Independent predictors for delayed entry were feeling healthy (aOR 3.7, 95% CI 1.5–9.1), first positive HIV test at other site (aOR 2.9, CI 1.2–7.1), history of injection drug use (IDU) (aOR 2.9, 95% CI 1.1–7.9), work/school conflicts (aOR 4.3, 95% CI 1.7–10.8), prior registration at another clinic (aOR 77.4, 95% CI 8.6–697), detention or imprisonment (aOR 10.3, 95% CI 1.8–58.2), and perceived distance to clinic (aOR 3.7, 95% CI 1.0–13.7).

**Conclusion:**

Delayed entry into HIV care in Vietnam is common and poses a significant challenge to preventing AIDS and opportunistic infections, decreasing mortality, and reducing HIV transmission. Improved linkages between testing and care are needed, particularly for patients who feel healthy, as well as incarcerated and drug-using populations who may face structural and social barriers to accessing care.

## Introduction

Delays between HIV diagnosis and registering for care may lead to late antiretroviral treatment (ART) initiation and increased morbidity and mortality. Early initiation of ART has been shown to improve long-term CD4 count recovery and immune restoration [1]–[3]. The impact of immune restoration has been clearly demonstrated in cohort studies and randomized clinical trials that have shown decreased mortality and morbidity, including AIDS defining clinical events and tuberculosis, with initiation of ART at higher CD4 counts [1], [4]–[12].

Decreasing the time from diagnosis to care registration and early initiation of ART for those eligible is a critical quality of care issue. People living with HIV (PLHIV) who delay entry to care not only lose the potential benefits of early ART to their own health but also risk transmitting HIV to others. Most patients with untreated HIV also have detectable virus in their blood and genital secretions, presenting the potential for transmission to their sexual and injecting partners. Early enrolment into care and ART for these populations can significantly reduce HIV transmission as part of a combined prevention strategy [4], [13]–[17]. Moreover, as larger segments of the HIV-infected population are enrolled and started on ART, transmission of HIV within the at-risk community can be significantly reduced [18], [19].

The reported level of attrition from diagnosis to enrollment in care varies widely across country programs and likely reflects differences in available resources, availability of data for measurement, and definitions of linkage, including time limits for successful linkage. As of July 2012, the Center for Disease Control (CDC) estimated that 20% of persons living with HIV (PLHIV) diagnosed in the United States have yet to enroll in care [20]. Similarly, two large provinces in India found attrition rates of 18 and 20% between diagnosis and registration [21]–[23]. A systemic review of 10 studies across sub-Saharan Africa found the median percentage of patients who were diagnosed and did not receive first CD4 cell results or clinical staging was 41% and ranged from 12 to 65% [24].

Many patients who enroll in care present at late stage of disease. Numerous studies in sub-Saharan Africa highlight the diverse array of factors associated with delays in seeking care, including geographic, social, economic, structural, and individual behavioral issues [25]–[29]. HIV programs across Asia likewise continue to identify late presentation for care and treatment initiation as a chronic HIV cascade of care challenge. A recent study in 22 sites in 13 Asian countries, including two sites in Vietnam, found that 36% of patients continued to present late with CD4 counts less than 200 cells/mm3 [8]. Studies conducted exclusively in Vietnam report findings similar to other Asian countries with concentrated HIV epidemics. In particular, social, structural, and individual behavioral factors, including alcohol and injection drug use, continue to influence late presentation to care and low utilization of health care services [30]–[38].

As of December 31^st^, 2012, there were an estimated 260,000 PLHIV in Vietnam, concentrated in the key affected populations (KAPs) of men who have sex with men (MSM), female sex workers (FSW), and injection drug users (IDU). Of these, 218,000 (84%) have been tested and diagnosed with HIV infection. Only 40% of women and 36% of men diagnosed with HIV are engaged in care and eligible for ART [39]–[41]. However, despite the rapid scale-up of ART with support from both the United States President’s Emergency Plan for AIDS Relief (PEPFAR) and the Global Fund for AIDS Tuberculosis and Malaria (GFATM), and an increase in the ART eligibility threshold to CD4<350 cell/mm^3^ in 2011, nearly half of all PLHIV across the nation continue to present late and initiate ART with CD4 counts <100 cells/mm^3^ [42]–[44].

The purpose of the study was to inform current programming and national HIV care quality improvement (HIVQual Vietnam) activities and to identify potential access and service quality issues at HIV OPCs that could be addressed to reduce delays in entry into care after HIV diagnosis. These findings will be helpful in addressing amenable access and service issues that result in unnecessary time lags from diagnosis to entering care and to ultimately reduce morbidity, mortality, and HIV transmission within the community.

## Methods

### Survey Design, Sites and Population

In Vietnam, HIV testing is available through stand-alone clinics or co-located with a network of 318 free public HIV outpatient clinics (OPCs) across the country. All PLHIV with CD4 count <350 or WHO clinical stage III/IV conditions are eligible for ART [43]. These services are primarily delivered at provincial and district levels and are supported through ongoing funding from PEPFAR, the GFATM, and the National HIV/AIDS Treatment Program.

This cross-sectional survey was conducted at one urban OPC in Ho Chi Minh City (HCMC) and two rural OPCs located in Can Tho and An Giang provinces, representing the three provinces with the highest burden of HIV infections in Southern Vietnam. Three sites were selected based on national HIV quality of care data that demonstrated high percentages (74–91%) of patients registering for ART with CD4 counts <250 cells/mm^3^.

The district 8 OPC is located in a densely populated urban district with an area of 19 km^2^ and a population of approximately 426,000 residents in HCMC, the largest city in Vietnam. The OPC was opened in June 2003 and was one of the first OPCs to provide ART in Vietnam with PEPFAR support. The clinic offers integrated methadone maintenance therapy (MMT), HIV testing and counseling (HTC), and HIV care and currently supports over 1,500 patients on ART. Thot Not OPC is located in a rural district of Can Tho province in the Mekong Delta. The district has an area of 117 km^2^ and a population of approximately 160,000 residents. The OPC is located within Thot Not District hospital. It provides integrated MMT, HTC, and ART services and currently supports 369 patients on ART. Tinh Bien OPC is a located in a rural district of An Giang province in the Mekong Delta along the border with Cambodia. The district covers a relatively large area of 337 km^2^ and has a population of approximately 113,000 residents. The OPC provides HTC and ART services and currently supports 489 patients on ART [45], [46].

Patients were eligible to participate if they were: adults ≥18 years old, had documented HIV infection, first registered at the OPC between July 1, 2012 and June 30, 2013, were not currently taking ART at the time of registration, and were late presenters, defined as first CD4 count at the OPC≤250 cells/mm^3^.

### Data collection

Potential participants were approached at routine monthly follow-up clinic appointments and invited to participate in the survey. The purpose of voluntary participation in the survey and its procedures, risks, and benefits were explained and all questions answered. All three sites received intensive training from FHI 360 staff on survey administration. Nurses and counselors were all trained on how to obtain verbal consent and administer the survey in an objective and nonjudgmental manner. The first 10 to 20 interviews at each site were observed by project managers to insure that the staff could administer the survey appropriately. Counselors used a standardized verbal consent script and all participants gave verbal informed consent, which was documented in a study log.

OPC staff administered a standardized survey questionnaire which included demographics, risk factors for HIV transmission, previous HIV treatment, and patient-centered reasons for delays in HIV testing and OPC registration. Reasons for delays included access issues (i.e., distance, administrative barriers), patient attitudes (i.e., feeling healthy, fear of stigma, desire to avoid medications) and patient perceptions of service quality. Patient-centered reasons for delayed testing and entry into care were rated on a 3-point Likert scale as having “no effect”, “some effect”, or “much affect.” For the bivariate and multivariate analyses each factor was converted to a binary scale of “no effect” or “any effect” by combining the last two categories in the Likert scale.

All patients were asked the month and year of first HIV positive test. If supporting documentation was available in the medical chart, the exact date of the positive HIV test result was recorded. Otherwise, the date of first HIV positive test was recorded as the first day of the following month after the patient-reported month of diagnosis. Date of OPC registration, date of ART initiation, and first CD4 count were obtained from clinic medical records. Participants received no compensation for participation.

### Statistical Analysis

Delayed entry to care was defined as >3 months from the date of the first positive confirmed HIV test to registration at the OPC. Correlates of delayed entry to care were identified using a t-test for continuous variables and chi-square or Fisher’s exact test for categorical variables. Reported reasons for delayed entry to care were also stratified based on previous enrollment in another OPC and further analyzed for differences using chi-square or Fisher’s exact tests. Multivariate logistic regression was conducted using the backward stepwise method to determine independent predictors for delayed entry to care. All variables significant at p<0.10 on bivariate analysis were entered into the multivariate model, which was controlled for sex and age. Goodness-of-fit for the final model was assessed using the Hosmer and Lemeshow test. All reported p-values were two-tailed, and p-value<0.05 was considered statistically significant. Data analysis was completed on SPSS version 22 (IBM, Armonk, New York, USA).

### Ethics Statement

Verbal consent was obtained from all participants before enrolling in the survey. All data collection instruments were anonymous; no identifying information such as name, address, telephone number, birth date, or medical record number was recorded. The only link between patients and the data was a paper-based key at each location that listed medical record numbers and study subject numbers. The key at each location was stored separately from study data in a locked drawer and was destroyed after all data was collected and before data analysis occurred.

The protocol was reviewed by the institutional review board of FHI360 in Research Triangle Park, North Carolina, USA and determined to be exempt from full board review as a public health improvement activity. The project was also approved by the Departments of Health in each of the three collaborating provinces.

## Results

### Characteristics of study population

One hundred ninety six participants completed the survey, representing 96% (196/204) of the OPC patients screened and deemed eligible. Of the 8 patients that did not participate, 2 patients died prior to date of interview, 2 missed appointments during the interview period, and 4 declined to give consent.

Demographics and other characteristics of the survey sample are shown in Table 1. The majority of participants were male (61%), one-third (33%) had history of injection drug use (IDU), 18% had previously received care at another OPC, and 13% had previously taken ART. Most prior ART had been accessed in the public sector; only one patient reported previously receiving ART outside of the public system.

**Table 1:** Characteristics of participants registered into care in Vietnam with first CD4<250 cells/mm^3^ at time of enrollment.

Characteristics	Earlier entry(< = 3 months)	Delayed entry(>3 months)	Total
	N = 107	N = 89	N = 196
	n (%)	n (%)	n (%)
Age (Median, years)	34	33	33.5
CD4 count (Median,cell/mm^3^)	73	65	68
Gender			
Male	69 (64)	51 (57)	120 (61)
Female	38 (36)	38 (43)	76 (39)
Location			
Urban	53 (50)	62 (70)	115 (59)
Rural	54 (50)	27 (30)	81 (41)
Risk factor fortransmission*			
History of IDU	20 (19)	44 (49)	64 (33)
Heterosexual sex	85 (79)	48 (54)	133 (68)
MSM	2 (2)	1 (1)	4 (2)
Place of first HIVpositive test			
At this OPC	59 (55)	33 (37)	92 (47)
Other site	48 (45)	56 (63)	104 (53)
Ever register at other OPC			
No	106 (99)	54 (61)	160 (82)
Yes	1 (1)	35 (39)	36 (18)
Ever take ARVfrom other site			
No	106 (99)	64 (72)	170 (87)
Yes	1 (1)	25 (28)	26 (13)
Any delay in gettingthe HIV test?			
No	24 (22)	47 (53)	71 (36)
Yes	83 (78)	42. (47)	125 (64)

*IDU, injection drug use; MSM, men who have sex with men; OPC, outpatient clinic.*

*participants could report more than one risk factor for transmission.

The median CD4 at entry to care was 68 cells/mm^3^ (interquartile range (IQR) 22–154 cells/mm^3^) and 62% of participants had CD4<100 cells/mm^3^. By definition, all participants met eligibility criteria for ART through the national HIV treatment program.

During the interview, participants were first asked if they had delays in getting tested for HIV. Seventy-three percent (n = 143) of participants reported delays in seeking HIV testing. All but one patient reported a time lag from testing to registration of at least one day. Forty-five percent of participants (n = 89) met the definition for delayed entry to care (>3 months since first confirmed positive HIV test), and 38% were delayed in entry to care by more than 180 days. Once enrolled, the median time from OPC registration to ART initiation was 19 days (IQR 9–33 days) with 72% and 92% initiating ART by 30 and 60 days, respectively.

Those participants who reported delay in HIV testing (n = 143) were asked about factors that affected getting tested (Table 2). The most common reasons reported by participants for delayed testing were feeling healthy (71%), fear of stigma and discrimination in the community (43%), time conflicts with work or school (31%), did not want to know if infected (30%), and fear of lack of confidentiality (27%).

**Table 2:** Reasons for delay in HIV testing among patients in Vietnam (n = 143).

Reasons	Patients reporting n (%)
Felt healthy andthought the test was not necessary	101 (71)
Fear of stigma anddiscrimination in community	61 (43)
Had to work or go to school	44 (31)
If HIV infected, didnot want to know	43 (30)
Fear the HIV test result wasnot confidential	39 (27)
Did not know the locationfor HIV testing	36 (25)
Afraid of thecost/did not have money	26 (18)
Fear of stigma anddiscrimination at facility	19 (13)
Test center was too far away	12 (8.4)
Administration andformalities were too difficult	8 (5.6)
Detention or imprisonment	6 (4.2)
Fear of detention or imprisonment	5 (3.5)
Perception of lowservice quality at facility	2 (1.4)

Forty-five percent of participants delayed entry into care. The median CD4 count at entry to care for this group was 65 cells/mm3. The most common reasons reported by participants for delayed entry were feeling healthy (51%), fear of stigma and discrimination in the community (41%), time conflicts with work or school (33%), and fear of lack of confidentiality (26%)(Table 3). Patients who had previously registered at other clinics and those who were registering for care for the first time cited the same top three reasons for delayed entry into care: feeling healthy, fear of stigma and discrimination, and time conflicts. However, patients who had previously registered at other clinics were more likely to cite not wanting to take medicine, detention or imprisonment and perception of low quality at the OPC as reasons for delayed entry to care.

**Table 3:** Reasons for delay in seeking HIV care after first positive HIV test in Vietnam (n = 196).

	Total	Prior registration in other OPC		
Reasons	n (%)	Yes (n = 36)	No (n = 160)	p-value
Felt healthy andthought the test/treatmentwas not necessary	99 (51)	22 (61)	77 (50)	0.23
Fear of stigma anddiscrimination in community	81 (41)	16 (44)	65 (43)	0.86
Had to work or go to school	64 (33)	17 (47)	47 (31)	0.06
Fear the HIV test result/treatmentwas not confidential	50 (26)	9 (25)	41 (27)	0.84
Fear of medication side-effects	39 (20)	10 (29)	29 (19)	0.20
Afraid of the cost/did not have money	30 (15)	5 (14)	25 (16)	0.73
Fear of stigma anddiscrimination at facility	29 (15)	5 (14)	24 (16)	0.80
Don’t want to take medicine for HIV	27 (14)	12 (33)	15 (10)	<0.01*
Don’t know the location of OPC	25 (13)	6 (17)	19 (12)	0.49
Test center/HIVoutpatient clinic was too far	18 (9.2)	4 (11)	14 (9)	0.71
Administration andformalities were too difficult	14 (7.1)	5 (14)	9 (6)	0.10
Detention or imprisonment	19 (9.7)	8 (22)	11 (7)	<0.01*
Fear of detention orimprisonment	8 (4.1)	2 (6)	6 (4)	0.66
Perception of low service quality at facility	5 (2.6)	3 (8)	2 (1)	0.02*

OPC, outpatient clinic; *p≤0.05.

The mean age of participants with delayed entry to care was 33.5 years, which was not statistically different than for those with earlier entry of less than 90 days (34.6 years; p = 0.19). There was also no difference in mean CD4 counts at ART initiation, 88 and 90 cells/mm^3^, for those with delayed entry and earlier entry, respectively (p = 0.85). No statistically significant difference was observed between women and men for delayed entry to care (p = 0.30). More than half (54%) of urban patients had delayed entry, compared to one-third (33%) of rural patients (p = 0.04).

Factors independently associated with delayed entry were analyzed using multivariate logistic regression. In the final multivariate analysis model, “prior registration at another OPC” was included while “prior ART use” was excluded because these two variables were highly correlated (25/26 participants who reported prior ART use also reported prior registration at another OPC). The results of univariate and multivariate analyses for factors associated with delayed entry after first positive HIV test are shown in Table 4. Result of the Hosmer and Lemeshow test was 0.780, indicating that the multivariate model fit the data. Independent predictors for delayed entry were 1) feeling healthy (aOR 3.7, 95% CI 1.5–9.1), 2) first HIV positive test at other site (aOR 2.9, CI 1.2–7.1), 3) history of IDU (aOR 2.9, 95% CI 1.1–7.9), 4) work/school conflicts (aOR 4.3, 95% CI 1.7–10.8), 5) prior registration at another clinic (aOR 77.4, 95% CI8.6–697), 6) detention or imprisonment (aOR 10.3, 95% CI 1.8–58.2), and 7) perceived distance to clinic (aOR 3.7, 95% CI 1.0–13.7).

**Table 4:** Factors associated with delayed entry to care after HIV Diagnosis among late presenters (n = 196).

Factor		Late Entryn (%)	OR (95% CI)	p-value	aOR(95% CI)	p-value
		n = 89				
Age (mean)		33.5 years		0.19		
Sex	Female(n = 76)	38 (50)				
	Male (n = 120)	51 (43)	0.74 (0.42–1.3)	0.30		
IDU	No (n = 132)	45 (34)				
	Yes (n = 64)	44 (69)	4.3 (2.2–8.1)	<0.01	2.9 (1.1–7.9)	0.03
Domicile location	Urban (n = 115)	62 (54)				
	Rural (n = 81)	27 (33)	0.43(0.24–0.77)	0.04		
Location of first	At OPC(n = 92)	33 (36)				
positive HIV test	Other (n = 104)	56 (53)	2.1 (1.2–3.7)	0.01	2.9 (1.2–7.1)	0.02
Registered first at	No (n = 160)	54 (34)				
other OPC	Yes (n = 36)	35 (97)	68.7 (9.2–515)	<0.01	77.4 (8.6–697)	<0.01
History of previous	No (n = 170)	64 (38)				
ART	Yes (n = 26)	25 (96)	41.4 (5.5–313)	<0.01		
Felt Healthy	No (n = 91)	26 (29)				
	Yes (n = 99)	63 (64)	4.4 (2.4–8.1)	<0.01	3.7 (1.5–9.1)	0.01
Fear stigma and	No (n = 107)	47 (44)				
discrimination in	Yes (n = 81)	41 (51)	1.3 (0.7–2.3)	0.36		
the community						
Work/school time	No (n = 126)	45 (36)				
conflict	Yes (n = 64)	44 (69)	4.0 (2.1–7.5)	<0.01	4.3 (1.7–10.8)	<0.01
Fear loss of	No (n = 140)	62 (44)				
Confidentiality	Yes (n = 50)	27 (54)	1.5 (0.8–2.8)	0.24		
Fear side effects of	No (n = 150)	69 (46)				
medicine	Yes (n = 39)	19 (49)	1.2 (0.6–2.3)	0.76		
Fear cost of	No (n = 160)	73 (46)				
treatment	Yes (n = 30)	16 (53)	1.4 (0.6–3.0)	0.44		
Fear stigma and	No (n = 161)	77 (48)				
discrimination in OPC	Yes (n = 29)	12 (41)	0.8 (0.4–1.7)	0.52		
Don’t want to take	No (n = 163)	68 (42)				
medicine	Yes (n = 27)	21 (78)	4.9 (1.9–12.8)	<0.01		
Detention/prison	No (n = 171)	73 (43)				
	Yes (n = 19)	16 (84)	7.2 (2.0–25.4)	0.01	10.3 (1.8–58.2)	0.01
OPC too far away	No (n = 172)	76 (44)				
	Yes (n = 18)	13 (72)	3.3 (1.1–9.6)	0.02	3.7 (1.0–13.7)	0.05

*OR, odds ratio; aOR, adjusted odds ratio; IDU, injection drug user; ART, antiretroviral therapy; OPC, outpatient clinic.*

## Discussion

In this study we found high rates of delayed testing and delayed entry into HIV care after diagnosis across the selected clinics. The most common reason reported by patients for both delaying testing and enrollment to care after testing was that they “felt healthy.” Patients who reported feeling healthy were more than three times as likely to have delayed entry into care. This phenomenon has also been reported in other studies. In New York City, the lack of perceived need for medical care was most commonly cited, after denial of HIV status, among patients who failed to engage into care within three months [47]. Likewise, Thanawuth, et al. found that feeling healthy was the leading reason why patients in southern Thailand delayed entry into HIV care after diagnosis and that of those who engaged late, 91% only entered care after developing symptoms [30].

Fear of loss of confidentiality in the clinic setting and fear of stigma or discrimination in the community were each reported by more than a quarter of participants. This suggests that stigma continues to be a lingering issue among PLHIV in Vietnam. These fears may reflect past national prevention campaigns that associated HIV with “social evils” and increased stigmatization of both KAPs and PLHIV [31]–[33], [35].

On multivariate analysis our study found independent associations between delayed entry with HIV testing at other sites, previous care at another OPC, detention/imprisonment, work and school conflicts, and perceived distance to the OPC. All of these factors represent potential procedural and structural access barriers that need to be addressed.

Donor funding for free voluntary HIV-testing clinics in Vietnam is declining and thus it is likely that the percentage of patients first testing positive for HIV outside of the current donor-supported public system will increase. This highlights the importance of coordination between health services and effective referral systems from other departments, institutions, and community-based testing sites to HIV care for early ART initiation. Improved referral systems may require the adaptation of modalities that have been proven to improve referral success rates in other settings, such as SMS adherence messages [48]–[50], active case management [51]–[54], and tracking systems that are coupled with in-person navigators [52]–[55].

The quality of pre- and post-test counseling can have a significant impact on early uptake of treatment after diagnosis [30], [32], [51], [53], [56]. Our survey findings highlight a number of key messages to patients and providers that may need to be emphasized in post-test counseling. Most importantly, counseling should focus on the benefits of early HIV treatment, before symptoms appear, to prevent morbidity and reduce transmission to sexual and injection use partners. Other important elements of post-test counseling include framing heroin abuse and HIV as treatable medical conditions rather than as “social evils,” confidentiality of HIV test results and treatment status, availability of free once daily ART regimens with fewer side effects than previously used drugs, location of OPCs, and referral to peer or community-based support groups for PLHIV.

Previous enrollment in another OPC was highly associated with delayed entry into care. About 18% of participants were previously registered at other OPCs; two-thirds of these patients received free ART from these OPCs before dropping out of care. It is not clear whether these patients were lost to follow-up (LTFU) and eventually migrated to a new OPC, or were unsuccessful transfers from other OPCs. Regardless, these patients reported similar reasons for delayed entry to care as patients who registered for the first time at an HIV clinic, with a few exceptions. They were more likely to report perceptions of lower service quality, unwillingness to take medicine, and detention or imprisonment, all of which may reflect why they originally disengaged from care. These findings reaffirm the importance of effective referral systems between HIV OPCs and active outreach and case tracking after LTFU [51], [54], [57].

A history of detention or incarceration and history of IDU were both significantly associated with delayed entry in care. Currently, persons with IDU history are often detained in government sponsored “06” drug rehabilitation centers or incarcerated in prison for drug related crimes. Access to HIV testing and care is extremely limited in these settings and as a result PLHIV must often wait for release to receive either. Referrals after release are not routine, leading to additional delays to entry into care [32]. However, injection drug use was independently associated with delayed entry to care, suggesting that other factors in addition to detention and incarceration are barriers to entry into care for persons who inject drugs.

Work/school conflicts and distance to OPC represent tangible access barriers to care and were also significantly associated with delayed entry into care. Currently in Vietnam, ART is primarily delivered at provincial and district level outpatient facilities that are open Monday through Friday, making both travel time and hours of operation a barrier to care, especially among employed or studying patients. Perceived distance to OPCs can be significant for patients. An expenditure analysis conducted across three Vietnamese provinces that included provincial hospitals and district health centers estimated that 28% of total outpatient care service delivery costs were related to transportation [58]. Ongoing decentralization of HIV care to the commune level that offers services throughout the week would likely improve patient convenience, linkage, and retention in care [32], [59].

This study has some important limitations. We did not specifically ask what triggered decisions to test for HIV or to enroll in care. Participants were enrolled based on presence in care, recorded CD4 count, and return for a routine monthly clinic visit. As a result, our study may underestimate delays in care because we could not include those patients who had not yet registered in care, died prior to registering in care, or did not obtain a CD4 count. In addition, unless the first HIV positive test was documented in the medical chart, the date of the first positive HIV test was based on patient self-report, which might not have been accurate.

Only three provinces and clinics with a relatively small sample size were included in the study, limiting ability to generalize these results to other provinces or countries. Patient experiences in these provinces may not be representative of other locations, particularly mountainous areas in Northern Vietnam where data is limited and patients may travel long distances to receive care. However, the clinics were a mixture of urban and rural sites across three provinces that account for nearly 60,000 PLHIV or approximately a quarter of the total estimated national HIV population [60]. Moreover, several factors found to be associated with delayed entry to care in this study were also reported in national and regional studies that examined factors for presentation at late-stage of disease [8], [30], [32].

Questionnaires were administered by OPC staff, potentially impairing the willingness of patients to report stigmatizing information, such as injection drug use status, detention/imprisonment history, or concerns surrounding quality or confidentiality of care. Although we did record IDU history we did not ask patients about alcohol abuse, which is increasingly seen among Vietnamese patients and is associated with poor adherence to care [36], [37].

## Conclusion

Delayed HIV testing and delayed registration into care after HIV diagnosis were common among participants in southern Vietnam. Many patients feel healthy despite low CD4 counts and may mistakenly believe they do not need testing or treatment until they are sick. Public health authorities must educate patients, providers across health services, and the community at large on the importance of confidentiality of test results and the benefits of early ART for both long-term health and reduced transmission to sexual and injection use partners. Public messaging campaigns can be reshaped to reduce stigma towards KAPs, particularly people who inject drugs. Decentralization of testing and care to the commune level and expanded access to HIV testing and care through outreach in the community and in closed settings would help ensure that PLHIV are identified and receive timely and continuous HIV treatment. Finally, a number of targeted interventions, including improvements in post-test counseling, CD4 counts performed at the point of HIV testing, patient navigators, and active case management to support linkage of patients to HIV services could be adapted to the Vietnam context to reduce delays in entry to care.

## Supporting Information

Informed Consent Script S1 **Informed consent in Vietnamese with English translation.**
(DOCX)

Questionnaire S1 **English translation of questionnaire.**
(DOC)

Questionnaire S2 **Original Vietnamese language questionnaire.**
(DOCX)
