# Supplementary material for: Risk Factors for Delayed Entrance into Care after Diagnosis among Patients with Late-Stage HIV Disease in Southern Vietnam
Source: PLoS One. 2014 Oct 16;9(10):e108939. doi: 10.1371/journal.pone.0108939 (PMC4199603; doi:10.1371/journal.pone.0108939)
Supplement: Informed Consent Script S1 — Informed consent in Vietnamese with English translation. (DOCX) [file pone.0108939.s001.docx]

**Factors Related to Delayed HIV Testing and Entrance**

**into Care among Late Presenters in Vietnam**

**Verbal Informed consent Script**

1. **Vietnamese**

**Hướng dẫn lấy đồng thuận của người tham gia phỏng vấn**

Xin chào! Tôi tên là:…, nhân viên của Phòng Khám Quận….Chúng tôi muốn tìm hiểu ý kiến của khách hàng về dịch vụ của phòng khám nhằm cải thiện tốt hơn. Chúng tôi thu thập thông tin từ khách hàng đang nhận dịch vụ tại Phòng khám….Chúng tôi sẽ sử dụng bảng hỏi để tìm hiểu ý kiến khách hàng. Bảng hỏi này giúp chúng tôi hiểu tại sao khách hàng đến đăng ký khám và điều trị trễ.

Sự tham gia của các anh chị là tự nguyện. Việc anh chị có tham gia hay không thì không hề ảnh hưởng gì đến việc chăm sóc điều trị tại đây.

**Khi anh chị tham gia phỏng vấn thì:**

- Anh/ chị sẽ trả lời các câu hỏi khoảng 15 phút
- Chúng tôi sẽ hỏi vài câu hỏi về HIV, các vấn đề xã hội, và có một vài câu có thể nhạy cảm
- Tên của anh/chị sẽ không được ghi vào bảng hỏi để đảm bảo tính bảo mật. Chúng tôi sẽ cố hết sức để đảm bảo bảo mật, tuy nhiên tôi không dám hứa 100%.
- Anh chị có thể từ chối tham gia. Hoặc trong lúc phỏng vấn, anh/chị có thể dừng bất cứ thời điểm nào hoặc bỏ qua bất kỳ câu hỏi nào

**Rủi ro có thể gặp phải**

Sự tham gia của anh chị có thể rủi ro. Có thể có vài câu hỏi tế nhị khiến anh chị không thoải mái.

**Quyền lợi**

Không có quyền lợi trực tiếp nào khi anh chị tham gia phỏng vấn

Sau khi lấy sự đồng thuận của khách hàng, nhân viên phỏng vấn điền thông tin vào phiếu sàng lọc và chấp thuận.

1. **English**

**Instruction to get verbal consent**

Good morning/ afternoon. My name is ……………..……., a staff member of OPC……………………….. We are here to collect information from clients to learn more about your ideas about our health service, in order to improve our intervention. We are collecting information from patients who receive care at ___________________ clinic. We want to ask them to provide their ideas about questions in the structural questionnaire to improve HIV care. This questionnaire will contain questions that help us understand reason why some clients come to clinic late for care & treatment.

Your participation in this part of the study is voluntary. Whether or not you decide to participate in this study will not affect your treatment and care at this clinic.

## If you decide to participate:

- You will answer the structural questionnaire around 15 minutes
- We will ask several questions about HIV health and social issues. We will also ask you a few questions that are sensitive to ask.
- Your name will not written in this form to ensure your confidentiality. We will do everything we can to keep your data secure, however, complete confidentiality cannot be promised.
- You do not have to join this study if you do not want to. If you do join, you can refuse to answer any questions, or to end the interview at any time without penalty.

**Possible risks**

Your participation in this part of the survey may involve risks. Some of the questions we ask may be of a personal nature, which may make you uncomfortable.

**Possible benefits**

There are no direct benefits to participating

After interviewee agree to participate, Interviewer begin to fill in the screening log to demonstrate that this patient agree to join in the interview voluntary.
