# Supplementary material for: Risk Factors for Delayed Entrance into Care after Diagnosis among Patients with Late-Stage HIV Disease in Southern Vietnam
Source: PLoS One. 2014 Oct 16;9(10):e108939. doi: 10.1371/journal.pone.0108939 (PMC4199603; doi:10.1371/journal.pone.0108939)
Supplement: Questionnaire S1 — English translation of questionnaire. (DOC) [file pone.0108939.s002.doc]

**QI tool Data Collection Form**

**Project: Quality Improvement Activity to Assess Reasons for Late Entry to Care in District 8 Outpatient Clinic in Ho Chi Minh City, Vietnam**

**Criteria to Participate in QI evaluation:**

1. **First register at Q8 OPC after July 01, 2012**
2. **First CD4 at Q8 OPC < 250 cells/mm**^3^
3. **Age > 18 years**

**Page 1: collect from patient medical chart**

| **Ref.** | **Information** |  | | |
| --- | --- | --- | --- | --- |
| 1 | QI Evaluation Code # |  | | |
| 2 | Age (years) | ...............years | | |
| 3 | Gender | Male ☐ | Female ☐ | |
| 4 | Date of patient registered at OPC | ........./………./………..  (*dd/mm/yy*) | | |
| 5 | First CD4 count at Q8 OPC (cells/mm^3^) | ...............cells/mm^3^ | | |
| 6 | Date of patient start ART at OPC | ........./………./………..  (*dd/mm/yy*)  ☐ check if patient did not start ART yet | | |
| 7 | What are the patients risk behaviors? | *Yes* | | *No* |
|  | Infectious drug user | ☐ | | ☐ |
|  | Unsafely sexual behavior | ☐ | | ☐ |
|  | MSM | ☐ | | ☐ |

**Page 2: collect from patient interview**

*Read this informed consent script before starting the interview.*

The district 8 OPC is conducting an evaluation to find out reasons why patients perfrom HIV testing and register late at the OPC when their CD4 count is already very low. The purpose is to improve the procedures at the OPC so that in the future patients will come at an earlier stage of HIV infection when the CD4 is higher. That way we can start ART for patients earlier and prevent infections and death in our patients. If you agree to participate then we will ask you some questions that will take 10-15 minutes. Your participation is voluntary and you can stop participation at any time. You can also refuse to answer any questions. Whether you participate or not and whether you answer all questions or not will not affect your care or treatment at the OPC. We **do not** write your name or medical record number on the data collection form and we will keep all of your personal information confidential to the best of our ability.

Do you agree to participate?

Allow the patient to ask any questions they have. Obtain verbal consent before proceeding to the next question.

| 8 | When was the first HIV test result that was positive you had? | ………./………..  (*mm/yy*) |
| --- | --- | --- |
| 9 | Where was the first positive HIV test you had? *(check only 1 answer)* | ☐ Q8 OPC  ☐ Pasteur Institute  ☐ Hospital for Tropical Diseases, Q5  ☐ other public hospital/clinic  ☐ other private hospital/clinic |
| 10 | Before coming to the Q8 OPC, did you ever register in another OPC? | ☐ YES ☐ NO |
| 11 | Before coming to the Q8 OPC, did you ever take ARV from another clinic? | ☐ YES ☐ NO |

| 12 | Was there any delay in getting the first HIV test that was positive? | ☐ NO DELAY: I had the first test soon after I thought about it or soon after it was recommended to me. *(If NO, skip to question 14 on next page)*  ☐ YES, there was a delay in getting the HIV test.  *(answer questions 12.1-12.14 and question 13 below)* | | | |
| --- | --- | --- | --- | --- | --- |
| ***Please answer how much affect each reason had on delaying the HIV test:***  ***much affect, little effect, or no effect.*** | | | | | |
| **Ref** | **Reasons** | | **Much affect** | **Less affect** | **No affect** |
| 12.1 | I did not know the location to be tested | | ☐ | ☐ | ☐ |
| 12.2 | The test center was too far | |  |  |  |
| 12.3 | Afraid of the cost/did not have money | | ☐ | ☐ | ☐ |
| 12.4 | Administration and formalities were too difficult | | ☐ | ☐ | ☐ |
| 12.5 | I felt healthy and thought the test was not necessary | | ☐ | ☐ | ☐ |
| 12.6 | Had to work or go to school and couldn’t make it to test center | | ☐ | ☐ | ☐ |
| 12.7 | Fear of Stigma & discrimination at test center | | ☐ | ☐ | ☐ |
| 12.8 | Fear of Stigma & discrimination in community | | ☐ | ☐ | ☐ |
| 12.9 | Fear that test HIV results were not confidential | | ☐ | ☐ | ☐ |
| 12.10 | Detention or imprisonment: could not test | | ☐ | ☐ | ☐ |
| 12.11 | Fear of detention or imprisonment if went to test center | | ☐ | ☐ | ☐ |
| 12.12 | If HIV infected did not want to know | | ☐ | ☐ | ☐ |
| 12.13 | Feel that quality of care or service at the test center was not good | | ☐ | ☐ | ☐ |
| 12.14 | Other write: | | ☐ | ☐ | ☐ |

1. What was the most important reason for this patient that delayed the HIV test?

………………………………………………………………………………………………………………………………………………………………………………………………………………………………………………………………………………………………

| 14 | ***Please answer how much affect each reason had on delaying coming to and registering at the OPC:***  ***much affect, little effect, or no effect.*** | | | |
| --- | --- | --- | --- | --- |
| **Ref** | **Reasons** | **Much affect** | **Less affect** | **No affect** |
| *14.1* | I did not know the location of the OPC | ☐ | ☐ | ☐ |
| *14.2* | The OPC was too far |  |  |  |
| *14.3* | Afraid of the cost/did not have money | ☐ | ☐ | ☐ |
| *14.4* | Administration and formalities were too difficult | ☐ | ☐ | ☐ |
| *14.5* | I felt healthy and thought that treatment was not necessary | ☐ | ☐ | ☐ |
| *14.6* | Had to work or go to school and couldn’t make it to test center | ☐ | ☐ | ☐ |
| *14.7* | Fear of Stigma & discrimination at OPC | ☐ | ☐ | ☐ |
| *14.8* | Fear of Stigma & discrimination in community | ☐ | ☐ | ☐ |
| *14.9* | Fear that test HIV status was not confidential | ☐ | ☐ | ☐ |
| *14.10* | Detention or imprisonment: could not go to OPC | ☐ | ☐ | ☐ |
| *14.11* | Fear of detention or imprisonment if went to OPC | ☐ | ☐ | ☐ |
| *14.12* | Did not want to take medicine for HIV | ☐ | ☐ | ☐ |
| *14.13* | Fear of side effects of medicines | ☐ | ☐ | ☐ |
| *14.14* | Feel that quality of care or service at the OPC was not good | ☐ | ☐ | ☐ |
| *14.15* | Other write: | ☐ | ☐ | ☐ |

1. What was the most important reason for this patient that delayed coming to the OPC?

………………………………………………………………………………………………………………………………………………………………………………………………………………………………………………………………………………………………

**THANK YOU FOR COMPLETING THE SURVEY**
