# Supplementary material for: Risk Factors for Delayed Entrance into Care after Diagnosis among Patients with Late-Stage HIV Disease in Southern Vietnam
Source: PLoS One. 2014 Oct 16;9(10):e108939. doi: 10.1371/journal.pone.0108939 (PMC4199603; doi:10.1371/journal.pone.0108939)
Supplement: Questionnaire S2 — Original Vietnamese language questionnaire. (DOCX) [file pone.0108939.s003.docx]

***PKNT: Quận 8***

**MẪU THU THẬP SỐ LIỆU VỀ CẢI THIỆN CHẤT LƯỢNG**

**Dự án: Hoạt động cải thiện chất lượng nhằm tìm ra nguyên nhân của việc đăng ký trễ vào chương trình chăm sóc điều trị tại phòng khám ngoại trú (PKNT) Quận 8, thành phố Hồ Chí Minh, Việt Nam.**

**Tiêu chuẩn tham gia đánh giá là những bệnh nhân:**

1. **Đăng ký lần đầu tại PKNT Quận 8 sau ngày 01/07/2012**
2. **Có xét nghiệm lần đầu tại PKNT Quận 8 có CD4<= 250 tế bào/mm3**
3. **Có độ tuổi >= 18 tuổi**

**Trang 1: Thu thập từ hồ sơ bệnh án**

| **STT** | **Thông tin cần thu thập** |  | | |
| --- | --- | --- | --- | --- |
| 1 | Số thứ tự |  | | |
| 2 | Tuổi bệnh nhân | ………..tuổi | | |
| 3 | Giới tính bệnh nhân | Nam ☐ | Nữ ☐ | |
| 4 | Ngày đăng ký chăm sóc điều trị tại PKNT | ..…../….…/…...  (ngày/tháng/năm) | | |
| 5 | Số lượng tế bào CD4 tại thời điểm đăng ký tại PKNT Quận 8 | ..………tế bào/ mm3 | | |
| 6 | Ngày BN bắt đầu điều trị ARV | ………./………./………  (ngày/tháng/năm)  ☐ Đã kiểm tra nếu bệnh nhân chưa điều trị ARV | | |
| 7 | Các hành vi nguy cơ của bệnh nhận | *Có* | | *Không* |
|  | Tiêm chích ma túy | ☐ | | ☐ |
|  | Tình dục không an toàn | ☐ | | ☐ |
|  | Tình dục đồng giới nam (MSM) | ☐ | | ☐ |

**Trang 2: Thu thập thông tin từ phỏng vấn bệnh nhân**

*Đọc phần thông báo này để lấy sự đồng thuận của bệnh nhân trước khi bắt đầu phỏng vấn.*

PKNT Quận 8 đang tổ chức một hoạt động để tìm hiểu nguyên nhân bệnh nhân đến đăng ký trễ tại phòng khám khi mà số lượng tế bào CD4 của họ đã rất thấp. Mục đích của hoạt động này là để cải thiện chất lượng quy trình chăm sóc điều trị tại PKNT quận 8 để sau này bệnh nhân đến đăng ký sớm hơn, khi mà số lượng tế bào CD4 của họ còn cao. Và với cách này, chúng ta có thể bắt đầu điều trị ARV cho bệnh nhân sớm hơn, giúp phòng các bệnh nhiễm trùng cơ hội và giảm tỷ lệ tử vong cho bệnh nhân. Nếu anh/ chị đồng ý tham gia thì chúng tôi xin phép phỏng vấn anh chị trong vòng 10-15 phút. Sự tham gia của anh/chị là tự nguyện nên anh /chị có thể dừng lại tại bất cứ thời điểm nào. Anh/ chị cũng có thể từ chối trả lời bất cứ câu hỏi nào. Việc anh chị có tham gia hay không, có trả lời tất cả các câu hỏi hay không thì hoàn toàn không ảnh hưởng gì đến việc chăm sóc và điều trị của anh/chị tại PKNT Quận 8. Chúng tôi **không viết tên** và mã hồ sơ bệnh án của anh chị vào mẫu thu thập thông tin và chúng tôi sẽ hết sức bảo mật thông tin cho các anh/chị.

Vậy, anh/chị có đồng ý tham gia không?

Bệnh nhân được quyền hỏi bất cứ câu hỏi nào. Lấy sự đồng thuận của bệnh nhân trước khi phỏng vấn.

| 8 | Anh/chị xét nghiệm HIV dương tính lần đầu tiên khi nào? | | ……………/……………….  (tháng/năm) | | | |
| --- | --- | --- | --- | --- | --- | --- |
| 9 | Anh/chị xét nghiệm HIV đầu tiên dương tính được làm ở đâu? | | ☐ PKNT Quận 8  ☐ Viện Pasteur  ☐ Bệnh viện nhiệt đới, Quận 5  ☐ Bệnh viện/ phòng khám công  ☐ Bệnh viện, phòng khám tư | | | |
| 10 | Trước khi đến PKNT Quận 8, anh/chị có đăng ký ở phòng khám khác chưa? | | ☐ có ☐ Không | | | |
| 11 | Trước khi đến PKNT Quận 8. Anh/ chị đã từng uống thuốc ARV ở đâu chưa? | | ☐ có ☐ Không | | | |
| 12 | Có yếu tố nào làm trì hoãn việc anh/chị làm xét nghiệm HIV lần đầu không? | | ☐ Không. Tôi đã xét nghiệm lần đầu ngay khi tôi nghi ngờ hoặc ngay khi tôi được khuyến khích làm. ( Nếu Không, hỏi đến câu 14 ở trang sau)  ☐ Có, có lý do để trì hoãn ( trả lời từ câu 12.1-12.14 | | | |
| **Vui lòng cho biết mức độ ảnh hưởng của các nguyên nhân dẫn đến việc xét nghiệm muộn: ảnh hưởng nhiều, ảnh hưởng ít, không ảnh hưởng** | | | | | | |
| **STT** | | **Lý do** | | **Ảnh hưởng nhiều** | **Ảnh hưởng ít** | **Không ảnh hưởng** |
| 12.1 | | Tôi không biết chỗ để xét nghiệm | | ☐ | ☐ | ☐ |
| 12.2 | | Chỗ xét nghiệm quá xa | | ☐ | ☐ | ☐ |
| 12.3 | | Sợ tốn tiền/ không có tiền | | ☐ | ☐ | ☐ |
| 12.4 | | Thủ tục hành chánh quá phức tạp | | ☐ | ☐ | ☐ |
| 12.5 | | Tôi cảm thấy khỏe và nghĩ xét nghiệm là không cần thiết (khi bệnh mới đi xét nghiệm) | | ☐ | ☐ | ☐ |
| 12.6 | | Phải đi làm/ đi học và không thể đến chỗ xét nghiệm | | ☐ | ☐ | ☐ |
| 12.7 | | Sợ kỳ thị và phân biệt đối xử tại nơi xét nghiệm | | ☐ | ☐ | ☐ |
| 12.8 | | Sợ cộng đồng kỳ thị và phân biệt đối xử | | ☐ | ☐ | ☐ |
| 12.9 | | Sợ kết quả xét nghiệm không được bảo mật | | ☐ | ☐ | ☐ |
| 12.10 | | Bị bắt hoặc ở tù nên không thể xét nghiệm | | ☐ | ☐ | ☐ |
| 12.11 | | Sợ bị bắt hoặc ở tù khi đến chỗ xét nghiệm | | ☐ | ☐ | ☐ |
| 12.12 | | Sợ ảnh hưởng đến cuộc sống khi biết mình bị nhiễm | | ☐ | ☐ | ☐ |
| 12.13 | | Cảm thấy chất lượng dịch vụ tại phòng khám không tốt | | ☐ | ☐ | ☐ |
| 12.14 | | Lý do khác: …………………………………… | | ☐ | ☐ | ☐ |

13. Lý do nào là ảnh hưởng nhiều nhất đến việc trì hoãn việc xét nghiệm của anh chị?

……………………………………………………………………………………………………………………………………………………………………………………………………………………………………………………………………………………………………………………………………………………………………………………………………………………………………………………………………………………………………………………………………………………………………………………………..

……………………………………………………………………………………………………………………………………………………………………………………………………………………………………………………………………………………………..

| **14** | **Vui lòng đánh giá mức độ ảnh hưởng của các nguyên nhân dưới đây lên việc đăng ký trễ tại PKNT Quận 8** | | | |
| --- | --- | --- | --- | --- |
|  | **Lý do** | **Ảnh hưởng nhiều** | **Ảnh hưởng ít** | **Không ảnh hưởng** |
| 14.1 | Tôi không biết địa chỉ PKNT | ☐ | ☐ | ☐ |
| 14.2 | Chỗ PKNT quá xa | ☐ | ☐ | ☐ |
| 14.3 | Sợ tốn tiền/ không có tiền | ☐ | ☐ | ☐ |
| 14.4 | Thủ tục hành chánh quá phức tạp | ☐ | ☐ | ☐ |
| 14.5 | Tôi cảm thấy khỏe và nghĩ xét nghiệm là chưa cần thiết | ☐ | ☐ | ☐ |
| 14.6 | Phải đi làm/ đi học và không thể đến PKNT sớm được | ☐ | ☐ | ☐ |
| 14.7 | Sợ kỳ thị và phân biệt đối xử tại PKNT | ☐ | ☐ | ☐ |
| 14.8 | Sợ cộng đồng kỳ thị và phân biệt đối xử | ☐ | ☐ | ☐ |
| 14.9 | Sợ việc điều trị tình trạng nhiễm HIV của mình không được bảo mật | ☐ | ☐ | ☐ |
| 14.10 | Bị bắt hoặc ở tù nên không thể đến PKNT | ☐ | ☐ | ☐ |
| 14.11 | Sợ bị bắt hoặc ở tù khi đến PKNT | ☐ | ☐ | ☐ |
| 14.12 | Không muốn uống thuốc để điều trị HIV | ☐ | ☐ | ☐ |
| 14.13 | Sợ tác dụng phụ của thuốc | ☐ | ☐ | ☐ |
| 14.14 | Cảm thấy chất lượng dịch vụ tại phòng khám không tốt | ☐ | ☐ | ☐ |
| 14.15 | Lý do khác: …………………………………… | ☐ | ☐ | ☐ |

1. Lý do nào là ảnh hưởng nhiều nhất đến việc trì hoãn việc đến đăng ký tại phòng khám của anh chị?

……………………………………………………………………………………………………………………………………………………………………………………………………………………………………………………………………………………………………………………………………………………………………………………………………………………………………………………………………………………………………………………………………………………………………………………………………………………………………………………………………………………………………………………

TP.HCM, ngày …..tháng…..năm 2013

Phỏng vấn viên

**XIN CHÂN THÀNH CẢM ƠN**
